# Investor attention on COVID-19 and African stock returns

**DOI:** 10.1016/j.mex.2020.101195

**Published:** 2020-12-29

**Authors:** Bernard Njindan Iyke, Sin-Yu Ho

**Affiliations:** a Centre for Financial Econometrics, Deakin Business School, Deakin University, Australia; b Department of Economics, College of Economic and Management Sciences University of South Africa, South Africa

**Keywords:** COVID-19, Coronavirus, Investor attention, Risk attitude, Stock markets, Stock returns, Africa

## Abstract

We examine the financial consequences of rising global investor attention or risk attitude related to the COVID-19 pandemic for African stock markets. Using daily investor attention indices, which are based on global COVD-19-related google search queries, and stock return indices for 14 African stock markets, we show that investor attention is an important determinant of stock returns. Our estimates suggest that an increase in investor attention consistently reduces stock returns in three stock markets, namely Botswana, Nigeria, and Zambia. In contrast, an increase in investor attention may enhance stock returns in Ghana and Tanzania. Our estimates imply that, in uncertain times like the current pandemic, stock markets like those of Ghana and Tanzania may offer potential diversification benefits to investors. We demonstrate that our estimates are broadly robust using a composite measure of investor attention.

• We built a direct and unambiguous measure of investor attention or risk attitude related to the COVID-19 pandemic.
• In an exponential generalised autoregressive heteroskedasticity of order one (i.e. EGARCH(1,1)) framework, we regressed stock returns on their first lags, investor attention, exchange rate returns, and commodity returns, and controlled for investor attention in the variance equation.

## Graphical abstract

**Figure:**
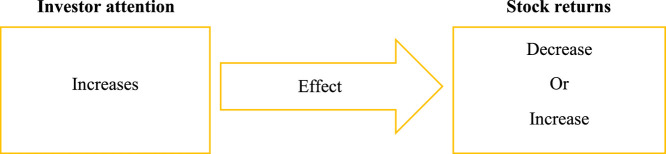

**Specifications Table**

**Table:** 

Subject Area	Economics and Finance
More specific subject area	*Behavioral Finance; Asset Pricing; Financial Econometrics; Predictive analytics*
Method name	• *Search Volume Index* • *Exponential Generalised Autoregressive Heteroskedasticity*
Name and reference of original method	• *Da, Z., Engelberg, J., & Gao, P. (2011). In search of attention. The Journal of Finance, 66(5), 1461-1499.* https://doi.org/10.1111/j.1540-6261.2011.01679.x • *Iyke, B. N. (2020a). COVID-19: The reaction of US oil and gas producers to the pandemic. Energy Research Letters, 1(2), 13912*. https://doi.org/10.46557/001c.13912.
Resource availability	• *Data is included in this publication.* • *Results can be reproduced using econometrics/statistical software like Gauss, R, Matlab, Stata, Eviews, Rats, and Python*

## Introduction

In this study, we examine the financial consequences of rising global investor attention related to the COVID-19 pandemic for African stock markets. Our empirical exploits reveal that investor attention related to the pandemic affects stock returns. Specifically, we find that: (a) an increase in investor attention reduces stock returns in three stock markets, namely Botswana, Nigeria, and Zambia; and (b) an increase in investor attention may enhance stock returns in Ghana and Tanzania. We show that our estimates are generally robust, using a composite measure of investor attention. Our findings are consistent with theoretical predictions that rising risk attitudes (or rising investor attention) during uncertain times may hurt stock returns, although some stock markets can be viable diversification options during such times. In turn, our estimates suggest that, in the current pandemic, stock markets like those of Ghana and Tanzania may offer potential diversification benefits to investors. Our estimations are based on measures of investor attention that we developed using global google search queries for keywords linked to the pandemic. We combine these investor attention measures with information on 14 stock return indices, exchange rates, and commodities over the period of 1 January 2020 to 26 November 2020.

Our study is motivated by the modern asset pricing models showing that attention influences asset prices [17]. Unlike these models, the classical asset pricing models assume that asset prices assimilate information instantaneously [3]. This means that investors allocate sufficient attention to assets, which contradicts reality because attention is a rare cognitive resource [13]. Thus, in theory, attention matters to asset prices. This prediction has been subjected to various empirical tests [1]. The main issues faced by empirical studies is that they do not have access to direct and unambiguous measures of attention. Thus, Da et al. [3] proposed a direct and unambiguous measure of attention based on aggregate google search frequency for keywords related to specific events. Inspired by their study, we build a direct and unambiguous measure of attention related to the COVID-19 pandemic in order to examine the financial consequences of investor attention on stock markets in Africa.

We are motivated to focus on African stock markets for various reasons. Despite the rapid growth of the literature on COVID-19 and financial markets [5,9,15,18], only two consider African stock markets (see [6,20]). Most African stock markets are relatively small and are dominated by a handful of large companies.^1^ Notwithstanding the relatively small sizes of most African stock markets, the continent has one of the largest stock exchanges in the world.^2^ This aside, African stock markets are less connected to global stock markets and could be alternative markets for global investors to diversify their risks. All these make understanding whether investor attention influences African stock markets, particularly during the pandemic, more appealing to us.

Our study contributes to the growing body of literature examining the impact of COVID-19 on financial markets. Most of these studies consider the impact of the pandemic on oil markets [5,9,15,18], stock markets ([19], Salisu and Sikiru, 2020), and foreign exchange markets [10,16]. None shows the impact of COVID-19-related investor attention on stock markets. Our study also contributes to the literature on investor attention and financial markets. This literature generally focuses on investor attention related to stocks [3], recessions, inflation, bankruptcy, financial crises, etc. [14]. We focus on investor attention related to the pandemic.

## Method details

### Results

We collected data on stock indices from 14 stock markets, namely Botswana, Egypt, Ghana, Kenya, Mauritius, Morocco, Namibia, Nigeria, South Africa, Tanzania, Tunisia, Uganda, Zambia and Zimbabwe, from Datastream. These are the leading stock markets in Africa.^3^ We followed the literature (see [2,7,11,12]) and collected data on exchange rates (local currencies per US dollar) and commodities because their returns are correlated to stock returns. The exchange rate data for Ghana, Morocco, Nigeria, Tanzania, and Tunisia were not available in Datastream, and hence were obtained from https://finance.yahoo.com/. For Zambia, we could not find data on spot exchange rates from both Datastream and https://finance.yahoo.com/. Therefore, we replaced the spot rates with the forward rates (bid price) from Datastream. We computed stock, exchange rate, and commodity returns as the logarithm changes in their price indices times 100, i.e. $r_{t}=lnY_{t}/lnY_{t-1}*100$, where $r_{t}$ and $Y_{t}$ denote returns and stock, exchange rate, and commodity indices at period t, respectively.

**Fig. 1:**
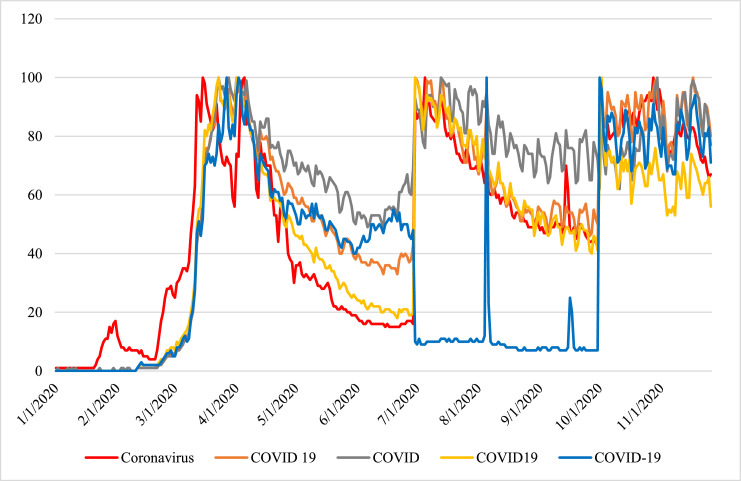
Trends in COVID-19-related google searches The figure shows the dynamics of our investor attention indicators based on google search frequency of COVID-19 related keywords/terms. During the earlier part of the pandemic, Coronavirus was the popularly searched term, while in the latter part, COVID became popular. The attention indicators range between zero and 100, with zero indicating lowest popularity and 100 indicating highest popularity of google search for the COVID-19-related keywords. The sample is from 1 January 2020 to 26 November 2020.

We measured investor attention by exploiting the powerful functionality of Google Trends (http://www.google.com/trends), which allows users to view the popularity of a given word over a period through the Search Volume Index. The index ranges from zero to 100; zero being the lowest and 100 being the peak popularity for a search query. Using Google Trends, we constructed investor attention related to the pandemic by searching for the main keywords/terms frequently used to describe the virus in the media and the literature, namely Coronavirus, COVID, COVID-19, COVID19, and COVID 19 (see [9]).^4^ Fig. 1, which shows the indices for each of these terms, suggests that during the early stage of the pandemic, Coronavirus was the most searched term, while in the latter stage COVID became the most searched. In our application, we measured rising investor attention as the logarithm changes in Coronavirus search query. To check for robustness, we constructed a composite measure of rising investor attention using principal component analysis of the logarithm changes in all five search queries.

In an exponential generalised autoregressive heteroskedasticity of order one (i.e. EGARCH(1,1)) framework, we regressed the stock returns on their first lags, investor attention, exchange rate returns, and commodity returns, and controlled for investor attention in the variance equation as follows:

(1) $$RET_{t}=\gamma_{0}+\gamma_{1}RET_{t-1}+\gamma_{2}IA_{t}+\gamma_{3}EX_{t}+\gamma_{4}COM_{t}+\varepsilon_{t}$$

where RET, IA, EX, and COM denote stock returns, investor attention, exchange rate returns, and commodity returns, respectively; $\gamma_{i}$ denote parameters of the mean equation; and $\varepsilon_{t}$ denotes the error term with a conditional variance, $\sigma_{t}^{2}$, of the form

(2) $$ln\sigma_{t}^{2}=\bar{\rho }+\alpha_{1}ln\sigma_{t-1}^{2}+\beta_{1}\left|\frac{\varepsilon_{t-1}}{\sigma_{t-1}}\right|+\tau_{1}\frac{\varepsilon_{t-1}}{\sigma_{t-1}}+\delta IA_{t}$$

where $\bar{\rho }$, $\alpha_{1}$, $\beta_{1}$, $\tau_{1}$, and δ denote parameters of the variance equation; ln and ∥ denote, respectively, the natural logarithm and absolute value operators.^5^

Table 1, which reports these estimates, shows that investor attention is an important determinant of stock returns in five stock markets in Africa, namely Botswana, Mauritius, Nigeria, Tanzania, and Zambia. Investor attention is negatively associated with stock returns in four of the stock markets (Botswana, Mauritius, Nigeria, and Zambia), and enhances stock returns in Tanzania. The negative effect of investor attention on stock returns is consistent with theoretical predictions.

**Table 1:** EGARCH(1,1) estimates of stock returns on investor attention.

Market	*RET*(-1)	*P*-value	*IA*	*P*-value	*EX*	*P*-value	*COM*	*P*-value
Botswana	0.0832	0.0031	−0.0008	0.0000	0.0264	0.0644	−0.0139	0.1223
Egypt	−0.0029	0.9699	−0.0007	0.9473	1.7712	0.0000	0.3755	0.0029
Ghana	−0.2800	0.0010	−0.0015	0.4948	0.0008	0.1103	0.0381	0.2911
Kenya	0.2887	0.0003	−0.0009	0.6813	−0.0098	0.8983	0.0854	0.0840
Mauritius	0.2602	0.0000	−0.0127	0.0003	0.1154	0.3028	0.9289	0.0000
Morocco	0.2023	0.0052	0.0053	0.1366	0.0234	0.5207	0.1178	0.0467
Namibia	−0.0029	0.9568	−0.0027	0.7562	−0.5600	0.0000	0.7199	0.0000
Nigeria	0.3169	0.0000	−0.0084	0.1758	−0.0257	0.5284	0.1546	0.0286
South Africa	0.0382	0.6014	−0.0035	0.4998	−0.1904	0.0107	0.5823	0.0000
Tanzania	−0.0159	0.4648	0.0031	0.0000	−0.0048	0.1482	0.0217	0.0000
Tunisia	0.3266	0.0000	0.0015	0.5387	−0.0788	0.1200	0.0113	0.6675
Uganda	0.4082	0.0000	0.0035	0.1860	−0.3868	0.0007	−0.3009	0.0000
Zambia	0.0046	0.9692	−0.0009	0.0074	−0.0352	0.1403	−0.0041	0.8099
Zimbabwe	0.3094	0.0000	−0.0002	0.9675	NA	NA	0.1556	0.1032

The table shows EGARCH(1,1) estimates of stock returns (*RET*) on lagged stock returns (*RET*(-1)), investor attention (*IA*) and controls (exchange rate returns (*EX*) and commodity returns (*COM*)). The variance equation controls for investor attention. We exclude exchange rate returns from the Zimbabwean regression because the local currency is unstable. NA denotes not applicable. The sample is from 1 January 2020 to 26 November 2020.

With regards to the other variables, exchange rate returns are an important determinant of stock returns in five stock markets (Botswana, Egypt, Namibia, South Africa, and Uganda). These estimates suggest that, in Namibia, South Africa, and Uganda, depreciation of the local currencies against the US dollar hurts stock returns, while it enhances stock returns in Botswana and Egypt. Studies on exchange rate exposure document that exchange rate depreciation may hurt or enhance stock returns [12]. Hence, both findings are consistent with the literature. In addition, commodity returns are statistically significant in nine of the 14 stock markets, meaning that commodity returns are a strong predictor of stock returns, which is consistent with the literature [11]. Commodity returns have a positive impact on stock returns in all nine but one stock market (Uganda). In African countries, there is a close positive correlation between commodity returns and economic growth [4]. Since economic growth is positively related to the stock market performance [8], our estimates for the eight stock markets are in line with these predictions. It confirms the theoretical prediction that stock and commodity returns tend to move in the same direction [7]. Similarly, the negative impact of commodity returns on stock returns for Uganda is consistent with the theoretical prediction that rising commodity returns are linked with rising levels of inflation and interest rates, and, in turn, bearish stock markets [2,11].

Since google searches about the pandemic were not only restricted to the word Coronavirus, as shown in Fig. 1, the estimates in Table 1 may not be showing the full picture. Accordingly, we constructed a composite indicator of investor attention by principal composite analysis using percentage changes in all five google scholar keyword searches, namely Coronavirus, COVID 19, COVID, COVID19, and COVID-19. Table 2 reports the EGARCH(1,1) estimates based on this composite investor attention index and shows that investor attention is an important predictor of five stock returns. Specifically, investor attention is negatively related to stock returns in Botswana, Nigeria, Tanzania, and Zambia, while it is positively related to stock returns in Ghana. Clearly, these estimates are consistent with the baseline for three stock markets, Botswana, Nigeria, and Zambia, and opposing in one, Tanzania. Overall, these estimates are consistent with our main finding, namely that investor attention is an important factor in stock returns.

**Table 2:** EGARCH(1,1) estimates of stock returns on the composite investor attention measure.

Market	*RET*(−1)	*P*-value	*CIA*	*P*-value	*EX*	*P*-value	*COM*	*P*-value
Botswana	0.0479	0.0000	−0.0077	0.0000	0.0575	0.0000	−0.0007	0.0000
Egypt	−0.0333	0.6411	−0.0859	0.1012	0.9676	0.0037	0.2867	0.0038
Ghana	−0.2765	0.0094	0.0486	0.0113	0.0009	0.0319	0.0549	0.1240
Kenya	0.3251	0.0000	−0.0255	0.5317	0.1602	0.2453	0.0644	0.3000
Mauritius	0.2688	NA	−0.4237	NA	−0.1431	NA	0.3454	NA
Morocco	0.2418	0.0022	0.0465	0.1314	0.0105	0.8118	0.1032	0.1060
Namibia	0.0025	0.9650	0.0264	0.7707	−0.6324	0.0000	0.7270	0.0000
Nigeria	0.2888	0.0000	−0.1251	0.0013	−0.0194	0.6482	0.1431	0.0422
South Africa	0.0476	0.5027	−0.0377	0.4716	−0.1912	0.0015	0.6848	0.0000
Tanzania	0.0377	0.6349	−0.0271	0.0019	−0.0397	0.1050	0.0015	0.8063
Tunisia	0.2933	0.0000	0.0040	0.8760	−0.1554	0.0184	0.0493	0.0991
Uganda	0.1992	0.0333	0.0205	0.6798	−0.5882	0.0204	−0.1911	0.0028
Zambia	−0.1513	0.0667	−0.0150	0.0000	−0.0603	0.0003	−0.0120	0.1254
Zimbabwe	−0.0793	0.7013	0.0533	0.7013	NA	NA	0.0881	0.5832

The table shows EGARCH(1,1) estimates of stock returns (*RET*) on lagged stock returns (*RET*(-1)), the composite investor attention measure (*CIA*) and controls (*EX* and *COM*). The variance equation controls for investor attention. We exclude exchange rate returns from the Zimbabwean regression because the currency is unstable. NA denotes not applicable. The sample is from 1 January 2020 to 26 November 2020.

## Concluding remarks

We examined the financial consequences of rising global investor attention related to the COVID-19 pandemic for African stock markets. To do this, we followed the literature and measured investor attention as global google search queries for keywords linked to the pandemic. Using this information, in addition to information on 14 stock return indices, exchange rates, and commodities, we showed that an increase in investor attention (or a rise in the popularity of global COVD-19-related google search queries) consistently reduces stock returns in three stock markets, namely Botswana, Nigeria, and Zambia. In contrast, an increase in investor attention may enhance stock returns in Ghana and Tanzania. These findings are consistent with theoretical predictions that rising risk attitudes (or rising investor attention) in uncertain times may hurt stock returns, although some stock markets may offer viable diversification options in such times. Our estimates therefore suggest that, in uncertain times like the current COVID-19 pandemic, stock markets like Ghana and Tanzania may offer potential diversification benefits to investors. We demonstrate that our estimates are broadly robust using a composite measure of investor attention.

## Appendix Supplementary materials

Image, application 1

## Footnotes

1 See https://www.un.org/africarenewal/magazine/august-2012/harnessing-african-stock-exchanges-promote-growth.

2 In 2013, the Johannesburg Stock Exchange (South Africa) recorded a market capitalization of approximately 1 trillion US dollars, making it the 19^th^ largest stock exchange globally. See https://www.jse.co.za/about/history-company-overview.

3 See https://www.cnbcafrica.com/financial/2020/01/03/the-best-and-worst-performing-african-stock-exchanges-in-2019/.

4 See https://edition.cnn.com/world/live-news/coronavirus-pandemic-12-01-20-intl/index.html and https://www.theguardian.com/world/live/2020/dec/01/coronavirus-live-news-us-may-begin-vaccinations-before-christmas-vietnam-sees-first-case-in-three-months for examples of media references to the virus.

5 See also Iyke [9], b] for a similar specification.
